# Editorial: Highlights in environmental psychology: pro-environmental purchase intent

**DOI:** 10.3389/fpsyg.2023.1268177

**Published:** 2023-09-05

**Authors:** Myriam Ertz, Lucian-Ionel Cioca, Luis F. Martinez

**Affiliations:** ^1^Laboratory of Research on New Forms of Consumption (LaboNFC), Department of Economics and Administrative Sciences, University of Quebec at Chicoutimi, Saguenay, QC, Canada; ^2^Industrial Engineering and Management Department, Faculty of Engineering, Lucian Blaga University of Sibiu, Sibiu, Romania; ^3^Nova School of Business and Economics, Universidade Nova de Lisboa, Carcavelos, Portugal

**Keywords:** pro-environmental purchase behavior, pro-environmental behavior (PEB), intentions, environmental psychology, responsible consumption, green product, sustainability, green consumption

Pro-environmental purchase is a topic of rising importance worldwide because it contributes to making consumption patterns more responsible (De Canio et al., 2021). Pro-environmental behavior can be defined as “behavior that harms the environment as little as possible, or even benefits the environment” (Steg and Vlek, 2009, p. 309; Ertz et al., 2016, p. 3971). Consequently, pro-environmental purchase (PEP) must be understood as a specific form of buying that harms the natural environment as little as possible and even benefits it. Products and services falling under that category are also called “green” and include, among others, energy-efficient household appliances (Nguyen et al., 2016; Teoh et al.), water-saving appliances (Wang and Tian), eco-tourism (Fennell, 2014), eco-friendly clothing (Wiederhold and Martinez, 2018), eco-designed products (Zeng et al., 2017), bioplastics-based products (Atiwesh et al., 2021), organic food products (Rodier et al., 2017), or products and services facilitating pro-environmental behaviors such as compost bags, for example. A key factor in this is consumers, who are a fundamental part of the overall consumption process and consumer society, and it is crucial to better investigate what drives them to pro-environmental purchases.

According to the selected studies, of the Research Topic, the ways to stimulate pro-environmental purchase intentions are very diverse.

The first and largest stream of papers in the Research Topic focuses on the impact of communication strategies. To Kim et al., the combination of narrative message style (storytelling) and two-sided messages increase (decrease) the perceived usefulness (skepticism) induced by pro-environmental messages on green products which in turn lead to greater behavioral intent. These results align with past results in classic marketing studies showing the effectiveness of two-sided ads in advertising, especially among Easterners (Ertz et al., 2021). Kim et al. emphasize the benefits of message-sidedness among Westerners and for promoting pro-environmental purchases specifically. Another classic technique to instill purchase intentions is comparative advertising and Ni et al. show that under certain circumstances (i.e., egoistic appeals and consumers with lower green involvement), comparative advertising strengthens consumers' purchase intentions of green products because such advertising leads to a higher perceived diagnosticity of information. From a self-construal theory perspective, Zheng et al. demonstrate that consumers with dependent self-construal exposed to green (vs. non-green) advertising appeals perceive a higher value in the green product and are thus more likely to pay a premium for green agricultural products. Wang et al.'s use of the stimuli-organism-response (SOR) model shows that organic appeals advertisements that provide information and knowledge about organic elements of a food product (e.g., health, safety, rich nutrition, and lack of chemicals), increase consumers' intentions to purchase organic milk. Intrinsic motives play a key role in that process because organic appeals spur intrinsic or autotelic motives that lead to higher purchase intentions. These results are partly corroborated by Lee's study showing that news consumption (about circular packaging in online shopping) positively affects environmental attitudes, subjective norms, perceived behavioral control, and habits which all subsequently influence intentions and thus mediate (i.e., explain) totally the effect of news consumption on behavioral intentions. Collectively, the results of these four studies show that the more consumers are exposed to relevant information about the impact of the product under consideration on the natural environment, the likelier they are to choose the pro-environmental option.

Another stream of research explores a wider range of marketing strategies and even situational factors to influence pro-environmental purchase intentions and provides a more nuanced perspective. Teoh et al. emphasize that the more extrinsic element of product pricing exerts the strongest effect on consumer purchase intentions (CPIs), followed by brand equity, while the psychological factor of environmental awareness—an intrinsic aspect—comes only third, and after-sales services have no influence whatsoever on CPIs. From a different perspective, Son et al. show how seven residential environment elements influence place dependence and place identity—two dimensions of place attachment—and that, at least, place identity influences not only satisfaction and word-of-mouth, but also pro-environmental behavior. Place dependence does not seem to impact pro-environmental behavior though. Affective response through place identity and satisfaction thus plays a key role in spurring pro-environmental behavior.

A third stream of studies focuses more extensively on the intra-psychic variables underlying pro-environmental purchase intentions. According to Wang et al., pro-environmental values and consumption values are both important sets of variables in that they further impact green purchase attitude, perceived behavioral control, and subjective norm, which are, respectively, the strongest contributors to green car purchase intentions. Meanwhile, the study by Wang and Tian shows that consumers might perceive some risk in pro-environmental options and that these are mainly functional, economic and psychological risks. Such perceptions have deleterious effects since they reduce consumers' quality trust and green trust in water-saving appliances, and indirectly impact willingness to buy through quality and green trust. Yet, in line with the first stream of research emphasizing the importance of knowledge (particularly Wang et al.; Lee), Wang and Tian show that consumer knowledge of water-saving appliances may weaken the negative impact of perceived risk on quality trust and green trust that indirectly inhibit purchase intentions. In sum, it is important to educate consumers with proper knowledge about the pro-environmental options so that they are better convinced about the appropriateness and benefits of those options.

In conclusion, these diverse studies illuminate further our understanding of the drivers and barriers to pro-environmental purchase intent, providing useful insights for both scholars and practitioners. The selected studies also pose a series of interesting questions that remain unanswered to further enhance pro-environmental purchase intentions, all of which could be tackled in future studies to extend the current research efforts.

## Author contributions

ME: Writing—original draft. L-IC: Writing—review and editing. LM: Writing—review and editing.
